# Inflammatory cytokines directly disrupt the bovine intestinal epithelial barrier

**DOI:** 10.1038/s41598-022-18771-y

**Published:** 2022-08-26

**Authors:** Charles K. Crawford, Veronica Lopez Cervantes, Mary L. Quilici, Aníbal G. Armién, María Questa, Muhammad S. Matloob, Leon D. Huynh, Aeelin Beltran, Sophie J. Karchemskiy, Katti R. Crakes, Amir Kol

**Affiliations:** Department of Pathology, Microbiology, and Immunology, School of Veterinary Medicine, University of California, Davis, Davis, CA USA

**Keywords:** Intestinal stem cells, Small intestine, Physiology, Cytokines, Inflammation

## Abstract

The small intestinal mucosa constitutes a physical barrier separating the gut lumen from sterile internal tissues. Junctional complexes between cells regulate transport across the barrier, preventing water loss and the entry of noxious molecules or pathogens. Inflammatory diseases in cattle disrupt this barrier; nonetheless, mechanisms of barrier disruption in cattle are poorly understood. We investigated the direct effects of three inflammatory cytokines, TNFα, IFNγ, and IL-18, on the bovine intestinal barrier utilizing intestinal organoids. Flux of fluorescein isothiocyanate (FITC)-labeled dextran was used to investigate barrier permeability. Immunocytochemistry and transmission electron microscopy were used to investigate junctional morphology, specifically tortuosity and length/width, respectively. Immunocytochemistry and flow cytometry was used to investigate cellular turnover via proliferation and apoptosis. Our study shows that 24-h cytokine treatment with TNFα or IFNγ significantly increased dextran permeability and tight junctional tortuosity, and reduced cellular proliferation. TNFα reduced the percentage of G2/M phase cells, and IFNγ treatment increased cell apoptotic rate. IL-18 did not directly induce significant changes to barrier permeability or cellular turnover. Our study concludes that the inflammatory cytokines, TNFα and IFNγ, directly induce intestinal epithelial barrier dysfunction and alter the tight junctional morphology and rate of cellular turnover in bovine intestinal epithelial cells.

## Introduction

The gastrointestinal (GI) tract comprises an extensive interface between an organism’s internal and external environments; it is responsible for various functions including nutrient digestion and absorption, hormone production, and it plays a primary role as a physical barrier between the luminal content of the GI tract and internal, sterile, tissues^1^. As a selective barrier with a primary goal of facilitating nutrient and water absorption, the GI tract must also prevent the entry of harmful substances/organisms, while also preventing the loss of water and beneficial solutes (ions, macromolecules etc.)^2^. The integrity of this barrier and the resulting permeability is influenced by numerous factors, one of note being inflammation. Human patients with varied inflammatory diseases such as eczema, psoriasis, pulmonary sarcoidosis, pancreatitis, and shock were found to have intestinal barrier disruption shown by differential sugar or 51Cr-EDTA absorption tests^3–6^. These clinical observations are supported by experimental data in mice^7,8^ and in human cell lines^9–12^ that indicate a direct role of inflammatory cytokines in intestinal barrier dysfunction. Systemic inflammation commonly affects cattle in modern dairy operations, and inflammatory diseases such as metritis, ruminitis, and mastitis as well as metabolic diseases, such as ketosis, negatively influence the health and wellbeing of the animals and result in reduced milk output, weight gain, reproduction, and survivorship^13–16^. Furthermore, there is a monetary cost of potentially up to $500 per affected animal including veterinary, labor, and reduced production costs^14^. Inflammatory diseases and other inflammatory stressors in cattle, such as heat stress and reduced feeding, coincide with disruption to the intestinal barrier^17–21^. While there is extensive focus on the inflammation caused by these diseases, it is not fully understood if the concomitant systemic inflammation induces intestinal barrier dysfunction in these cattle – a potentially underappreciated consequence that may be addressed to improve the livelihood and production of these agricultural animals.

The intestinal mucosa contains a continuous monolayer of specialized columnar epithelial cells that form a critical component of the barrier^22^. A pool of intestinal stem cells in the intestinal crypts proliferates and differentiates into specialized cell types to maintain the barrier as older cells slough off into the intestinal lumen^23^. The integrity of the epithelial cell surface is maintained by the junctional complexes, tight junctions, adherens junctions, and desmosomes which altogether regulate paracellular permeability^24^. Adherens junctions and desmosomes are more basolateral than tight junctions and form adhesive bonds that maintain the epithelial layer^25^. Tight junctions seal the intercellular space and are the most apical of the complexes; they contain the transmembrane proteins claudins and occludins, as well as regulatory proteins and the peripheral membrane proteins zonula occludens-1 (ZO-1) and zonula occludens-2 (ZO-2)^25^.

The mechanisms by which systemic inflammation may induce disruption of the intestinal barrier are multifaceted but not well understood in cattle. We have chosen to investigate the direct effects of three prototypical pro-inflammatory cytokines that are synthesized by three different cellular sources: epithelial cells (Interleukin-18), innate immune cells (tumor necrosis factor-α), and adaptive immune cells (interferon-γ)^26–28^. Interleukin-18 (IL-18) is produced by epithelial cells following inflammasome activation^29^ and induces the production of tumor necrosis factor-α (TNFα) and interferon-γ (IFNγ) in specialized immune cells^30^. It also acts as an autocrine/paracrine signal influencing intestinal goblet cell development, and inhibition of IL-18 signaling reduces mucus production and induces resultant mucosal damage in mice^31^. TNFα is a potent pro-inflammatory cytokine primarily produced by activated monocytes and macrophages^28^. IFNγ is produced by T lymphocytes and other specialized immune cells to aid in the adaptive immune response, especially in response to viral infection^27^. High levels of plasma TNFα and/or IFNγ have been recorded in multiple bovine inflammatory disorders such as metritis and rumenitis^15,32–35^. In in vitro epithelial monolayers—Caco2 and T84 human cell lines—treatment with TNFα and/or IFNγ disrupts the epithelial barrier as measured by transepithelial electrical resistance, bacterial translocation, or molecular flux^9–12^. Coinciding with the increased epithelial permeability, treatment with these two cytokines also altered the integrity and morphology of tight junction proteins^9,10^. Altogether, these three cytokines are tightly linked during the inflammatory process in vivo, and we utilized isolated in vitro treatments to determine their individual effects and contributions to inflammation-induced barrier disruption in the bovine gut.

We hypothesized that inflammatory cytokines directly disrupt the bovine intestinal barrier by altering tight junction structure and cell cycle. To further investigate these direct effects of inflammatory cytokines on intestinal barrier permeability in cattle, we utilize intestinal organoids (enteroids) derived from primary intestinal tissue as a relevant in vitro model^36^. We found that inflammatory cytokine treatment, specifically TNFα or IFNγ, increases the barrier permeability, increases junctional tortuosity, and disrupts epithelial cell cycle in bovine enteroids.

## Methods

Animals and Enteroid Generation: Tissue samples were obtained from cows according to a protocol approved by the Institutional Animal Care and Use Committee (IACUC) at UC Davis (protocol #21553). Tissues were collected from animals sacrificed for a separate study (IACUC protocol #21553) in accordance with American Veterinary Medical Association guidelines for the human slaughter of animals. No experiments in this study were conducted on live animals. Approximately 10 cm-long segments of jejunum and ileum were harvested from cows immediately post-mortem and placed in cold PBS with 25 μg/mL gentamicin (Thermo Fisher) and 100 U/mL penicillin/streptomycin (Gibco) and processed as previously described, with slight modifications described as follows^37,38^. The intestine was cut longitudinally and washed in PBS with gentamicin and penicillin/streptomycin. The mucosa was separated with a glass slide, minced with scissors, and placed into a 50 mL Falcon tube with cold PBS, gentamicin, and penicillin/streptomycin. The tube was shaken, and tissue fragments were allowed to settle before the supernatant was removed. This process was repeated until the supernatant was clear. Tubes were then centrifuged for 2 min at 200*g*, the supernatant was removed, and tissue was placed in a new 50 mL Falcon tube in 0.8 mM EDTA solution and incubated for 30 min at 4 °C with agitation to liberate intestinal crypts. After incubation, the tube was vigorously shaken, then centrifuged for 2 min at 400*g*. Supernatant was discarded, and the pellet was reconstituted in 25 mL of PBS, gentamicin, and penicillin/streptomycin. The tube was shaken, allowed to settle, and supernatant was passed through a 70 μm strainer into a new collection tube. This step was repeated. Tubes were centrifuged for 3 min at 100*g*, supernatant was removed, and crypts were reconstituted in DMEM/F12 media containing 1× B27 Supplement minus Vitamin A, 25 µg/mL gentamicin, and 100 U/mL penicillin/streptomycin. Crypts were then counted via a hemocytometer. The appropriate volume was extracted to plate 300 crypts per well. 20 μL of crypt solution was combined with 30 μL of ice-cold Matrigel™ (Corning) per well. 50 μL droplets were set in the center of a well of a pre-warmed 24 well plate. These domes were incubated at 37 °C for 10 min. 700 μL of Intesticult™ (Stemcell Technologies) growth media supplemented with 100 U/mL penicillin/streptomycin, 25 µg/mL gentamicin (only for primary culture), 100 mM Y-27632 (Tocris Bioscience), and 3 μM CHIR99021 (Tocris Bioscience). Y-27632 and CHIR99021 were used during initial organoid propagation and for the first 2–3 days after each passage. Media was changed every 3 days and organoids passaged mechanically every 7–10 days.

### Cytokine treatment

Enteroids were treated with 100 ng/mL of recombinant bovine TNFα (Invitrogen), IFNγ (Thermo Scientific), or IL-18 (Kingfisher Biotech) in Intesticult growth media on the fourth day following passage for a duration of 24 h. Following treatment, cytokine-treated growth media was removed, and organoids were prepared for downstream analysis.

### Immunofluorescence (IF)

Organoids were cultured in eight-well chamber slides (Thermo Scientific) for immunocytochemical staining. Media was removed and organoids were fixed in 4% paraformaldehyde (PFA) for 20 min at room temperature. Afterwards, organoids were permeabilized in PBS containing 0.5% Triton X-100 for 20 min at room temperature. Wells were washed in IF buffer (PBS containing 0.2% Triton X-10 and 0.05% Tween) and then blocked in IF buffer containing 1% BSA for 30 min at room temperature. After blocking, organoids were incubated in IF buffer containing 1% bovine serum albumin (BSA) with primary antibodies overnight at 4 °C. Organoids were then washed in IF buffer, then incubated in blocking solution with secondary antibodies for 1 h at room temperature. The following primary antibodies were used in this study: ZO-1 (Zo1-1A12, Invitrogen), Occludin (Polyclonal REF#71-1500, Invitrogen), Ki67 (SP6, Invitrogen) and Cleaved Caspase-3 (D175, Cell Signaling). Organoids were washed, then incubated in 0.1% DAPI in IF buffer for 10 min at room temperature. The gasket of the chamber slide was then removed, one drop of ProLong Gold antifade reagent (Invitrogen) was placed into well sections, and the slide was covered with a coverslip and allowed to cure for 24 h before being placed in 4 °C protected from light until imaging.

### FITC dextran permeability assay

Enteroids cultured in eight-well chamber slides were cultured in Intesticult growth media supplemented with 5 μg/mL 4 kDa or 70 kDa fluorescein isothiocyanate-dextran (FITC) for 1 h, then washed in PBS and imaged with a EVOS M5000 fluorescent microscope (Thermo Fisher), as modified from previous whole-enteroid FITC dextran permeability experiments^39,40^. FITC intensity inside of the enteroids (three data points per enteroid) was measured and normalized to FITC intensity outside of the enteroids (three data points averaged per enteroid) using an image analysis software (ImageJ).

### Cell cycle analyses

Enteroids were dissociated into a single cell suspension using TrypLE™ Express (Thermo Fisher) and a 40 μm cell strainer (Fisher Scientific). Cells were fixed in 70% ethanol and stained with the Muse Cell Cycle Kit according to the manufacturer’s instructions (Millipore, Hayward, CA). After staining, data was captured with a Muse Cell Analyzer (Millipore, Hayward, CA).

### Tortuosity analysis

Following immunofluorescent staining for the tight junction proteins, ZO-1 or Occludin, images were acquired using a TCS SP8 STED 3× confocal microscope (Leica Microsystems). Images were then processed utilizing the image analysis software (ImageJ) via a morphological segmentation plug-in, allowing for tortuosity calculation: the ratio of segment length and the Euclidian distance between the two points that define that given segment^41^.

### Electron microscopy

Enteroids were gently removed from wells using a cell scraper and cut P1000 micropipette tip and placed in Karnovsky’s fixative (3% glutaraldehyde and 2% formaldehyde in 0.1 M phosphate buffer, pH 7.4; all reagents were obtained from Electron Microscopy Sciences, Hatfield, PA, USA). Samples were submitted to the California Animal Health & Food Safety Laboratory (Davis, CA) for transmission electron microscopy processing, as previously described^42^. Briefly, organoids were postfixed with 1% osmium tetroxide in 0.1 M sodium cacodylate buffer and were dehydrated using a 25–100% ethyl alcohol gradient. Organoids were then infiltrated with 2:1 ethanol: EMbed 812 resin for 1 h and subsequently transferred to a 1:2 ethanol: EMbed 812 resin mixture for 1 h. Organoids were further infiltrated with 100% resin and were embedded and incubated at 58 °C for 24 h to polymerize the resin. Embedded samples were trimmed and sectioned on a Leica UC6 ultramicrotome (Leica Microsystems, Vienna, Austria). Thin Sects. (60–70 nm) were obtained and collected on a 200 mesh Nickle grid (Electron Microscopy Sciences, Hatfield, PA, USA). Grids were contrasted with 5% uranyl acetate for 20 min and Sato’s lead citrate for 6 min.All samples were visualized using a JEOL 1400 transmission electron microscope (JEOL LTD, Tokyo, Japan). Images were obtained and analyzed using a OneView camera system Model 1095, 16 megapixels with the Gatan Microscope Suite (GMS3.0) (Gatan Inc, Pleasanton, CA, USA). Tight junctions were identified via morphology, location, and electron density as described previously^43,44^.

### Statistical analysis

Experiments were conducted in three enteroid lines: C1, C2, and C3. Data collected from 4 kDa FITC imaging (N = 2505), Ki67 staining (N = 536), and Cleaved Caspase-3 staining (N = 653) for three different enteroid lines were nested and analyzed via nested ANOVA (α = 0.05) utilizing Holm- Šídák post hoc analysis. 70 kDa FITC (N = 5409) data were acquired from experiments conducted on two enteroid lines and analyzed via nested ANOVA (α = 0.05) utilizing Holm-Šídák post hoc analysis. Data from the 4 kDa FITC positive control experiment (untreated n = 120, EGTA n = 180) were analyzed via a one-tailed Mann–Whitney non-parametric test (α = 0.05). Data from ZO-1 (N = 3306) and Occludin (N = 2562) tortuosity analysis for one enteroid line were analyzed via Kruskal–Wallis non-parametric ANOVA (α = 0.05) utilizing Dunn’s post hoc analysis. Data from cell cycle analysis (N = 72) from three enteroid lines were aggregated and analyzed via two-way ANOVA (α = 0.05) utilizing Dunnet post hoc analysis. Values of tight junction lengths (N = 223) and widths (N = 237) collected via electron microscopy for two enteroid lines were aggregated and analyzed via Kruskal–Wallis non-parametric ANOVA (α = 0.05) utilizing Dunn’s post hoc analysis. Every experiment was conducted at least two times each.

## Results

### TNFα and IFNγ treatment disrupts normal enteroid morphology

**Figure 1:**
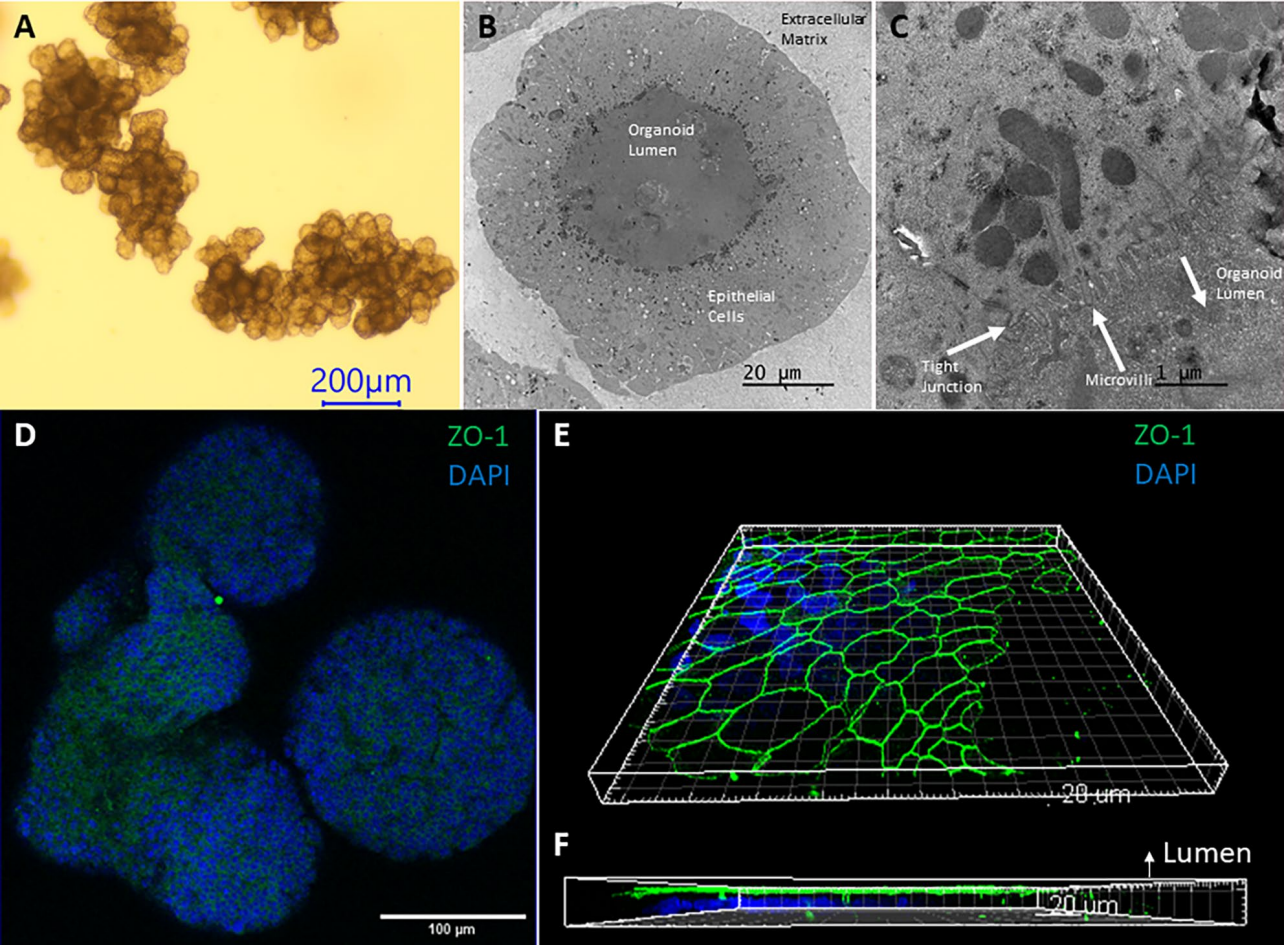
Bovine intestinal organoid characterization (**A**) Brightfield image of a bovine enteroid using a ×4 objective lens. Scale bar denotes 200 µm. (**B,C**) Transmission electron microscopy images of a bovine enteroid with annotated microvilli, tight junction, and lumen. (**D**) Whole mount Immunocytochemical imaging of ZO-1 (Alexa Fluor 488) and DAPI using a ×20 objective lens (**E,F**) 3D modeling (Imaris Image Software) of ZO-1 (Alexa Fluor 488) and DAPI staining using stacked confocal images taken with a ×100 objective lens, with annotated lumen, displaying the apical border of an enteroid.

Our bovine enteroids replicate the native gut micro-anatomy and display crypt-like domains with a central lumen (Fig. 1A). Low magnification (Fig. 1B) and high magnification (Fig. 1C) transmission electron microscopy (TEM) images of a bovine enteroid show microvilli protruding from enteroid cells towards to enteroid lumen confirming enterocyte cell identity. TEM further demonstrates apical tight junctions (Fig. 1C). ZO-1 staining (Fig. 1D) and 3D image analysis further demonstrates the apical positioning of the tight junctions (Fig. 1E,F).

**Figure 2:**
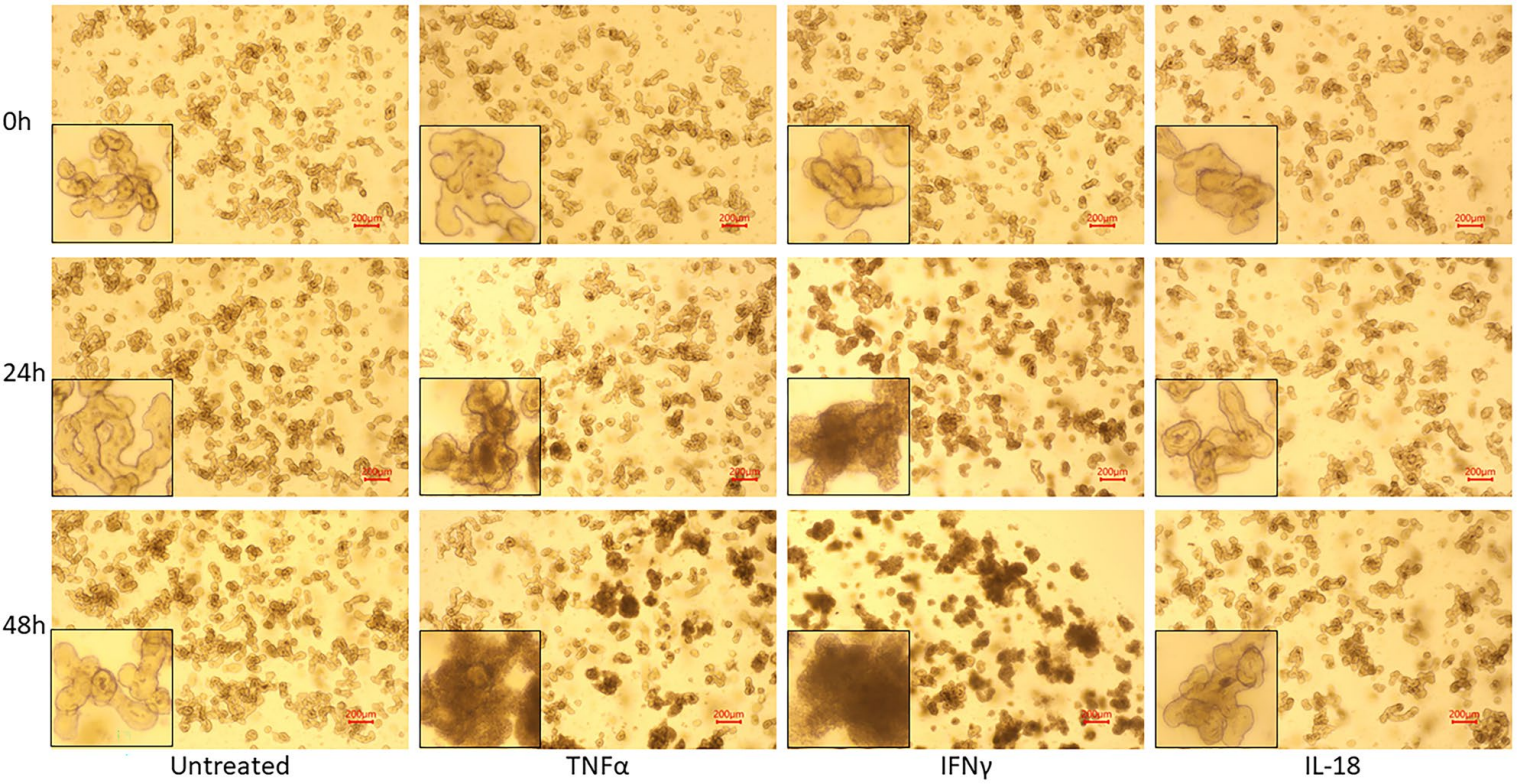
Morphological changes induced by cytokine treatment. Brightfield microscopy images of bovine intestinal organoids using a ×4 objective lens, treated with inflammatory cytokines for 24 and 48 h. Bottom left corners display a zoomed-in enteroid from each image. Scale bars denote 200 µm.

Bovine enteroids that were generated from 3 different cows, were treated with the inflammatory cytokine TNFα, IFNγ, or IL-18 at a concentration of 100 ng/mL for 24 and 48 h. Over the course of 48 h, untreated and IL-18-treated enteroids displayed unremarkable morphology consistent with extended enteroid culture: increased enteroid density, increased budding of enteroids, and slight darkening of the lumen (Fig. 2). However, enteroids treated with 100 ng/mL of TNFα or IFNγ displayed an increased darkening of the enclosed lumen, and enteroids treated with IFNγ also displayed irregular and ruffled basolateral margins. These morphological changes suggest an increased rate of dead cell accumulation within the enteroid lumen.

### TNFα and IFNγ treatment induces increased barrier permeability in bovine enteroids

**Figure 3:**
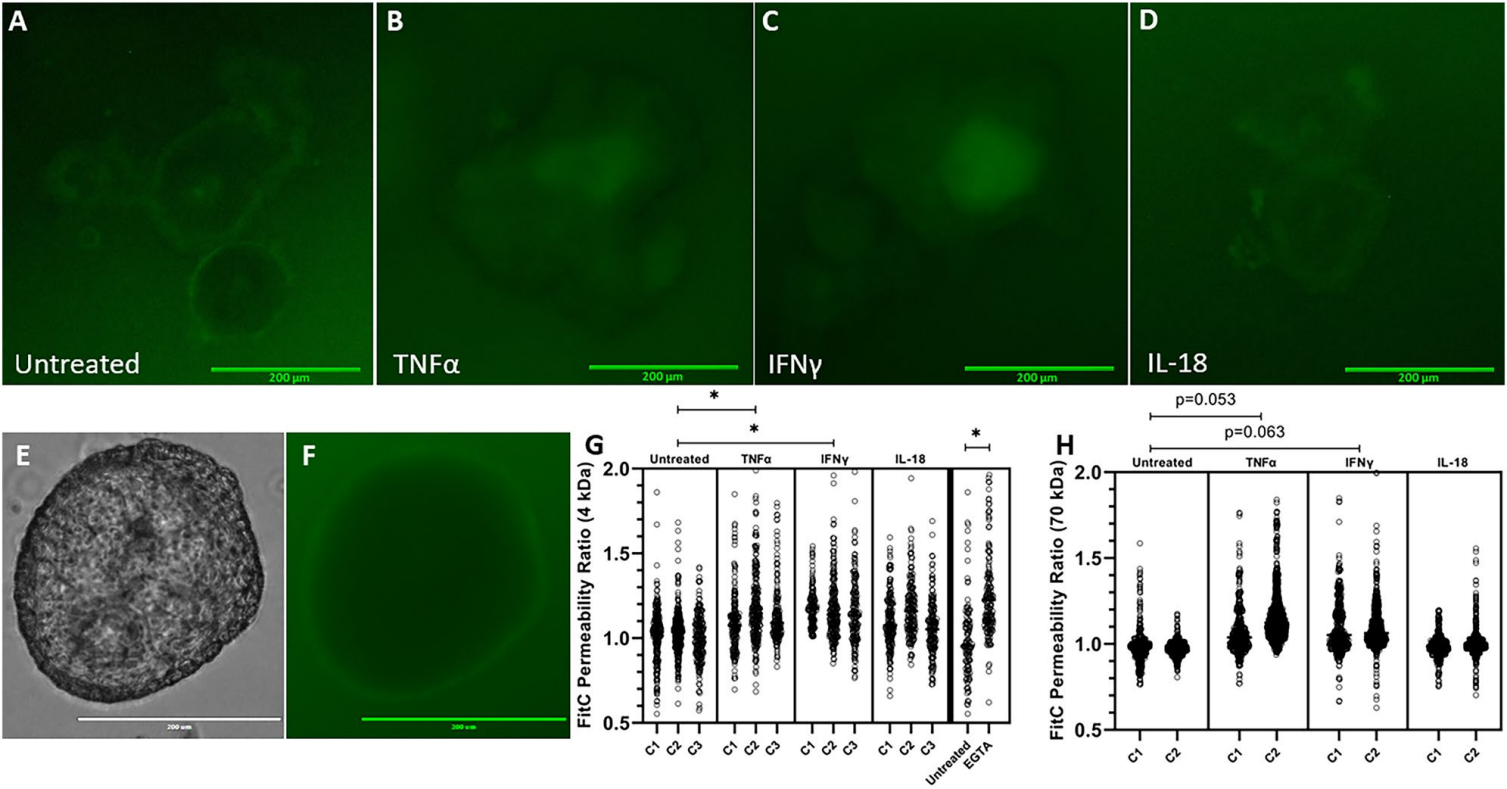
Cytokine treatment increases bovine intestinal organoid barrier permeability, as measured by FITC Dextran permeability. (**A–D**) Representative images of bovine intestinal organoids exposed to 4 kDa FITC Dextran following 24 h cytokine treatment acquired using a ×10 objective lens and GFP (470/525 nm ex/em) LED light cube. Scale bars denote 200 µm. (**E,F**) Representative images of an untreated enteroid following 70 kDa FITC Dextran exposure collected using a ×20 objective lens and brightfield or GFP LED light cube. Scale bars denote 300 µm. (**G,H**) Luminal FITC intensity normalized to external FITC intensity following 24 h cytokine treatment or two-hour treatment with 2 mM EGTA as a positive control. C1, C2, and C3 indicate individual enteroid lines. Nested ANOVA (α = 0.05) determined a significant effect of treatment was present for both 4 kDa and 70 kDa FITC Dextran (p = 0.0097 and p = 0.039, respectively). TNFα and IFNγ increase 4 kDa FITC Dextran permeability (p = 0.0257, p = 0.0132, respectively) as determined by Holm-Šídák post hoc analysis. EGTA, a positive control, increases FITC Dextran permeability (p < 0.0001) determined by one-tailed Mann–Whitney non-parametric test (α = 0.05). Holm-Šídák post hoc analysis for 70 kDa FITC Dextran displayed trending, but not significant, effects of TNFα and IFNγ treatment (p = 0.052 and p = 0.063, respectively).

We hypothesized that these deleterious morphologic changes are associated with increased permeability. To test our hypothesis and determine the underpinning mechanisms we determined the direct impact these inflammatory cytokines have on organoid dextran permeability utilizing a FITC dextran permeability assay (Fig. 3). 24-h cytokine treatment did induce a significant effect on 4 kDa barrier permeability (p = 0.0097); treatment of either TNFα or IFNγ increased the enteroid permeability to 4 kDa dextran and induced a significant increase in normalized FITC intensity relative to untreated enteroids (p = 0.0257, p = 0.0132, respectively) (Fig. 3G). 24-h cytokine treatment also induced a significant effect on 70 kDa barrier permeability (p = 0.039); TNFα or IFNγ trended towards inducing an effect (p = 0.053 p = 0.063, respectively) (Fig. 3H). There was no apparent effect of IL-18 treatment on 4 kDa or 70 kDa dextran permeability.

### TNFα and IFNγ treatment induces altered tight junction conformation

**Figure 4:**
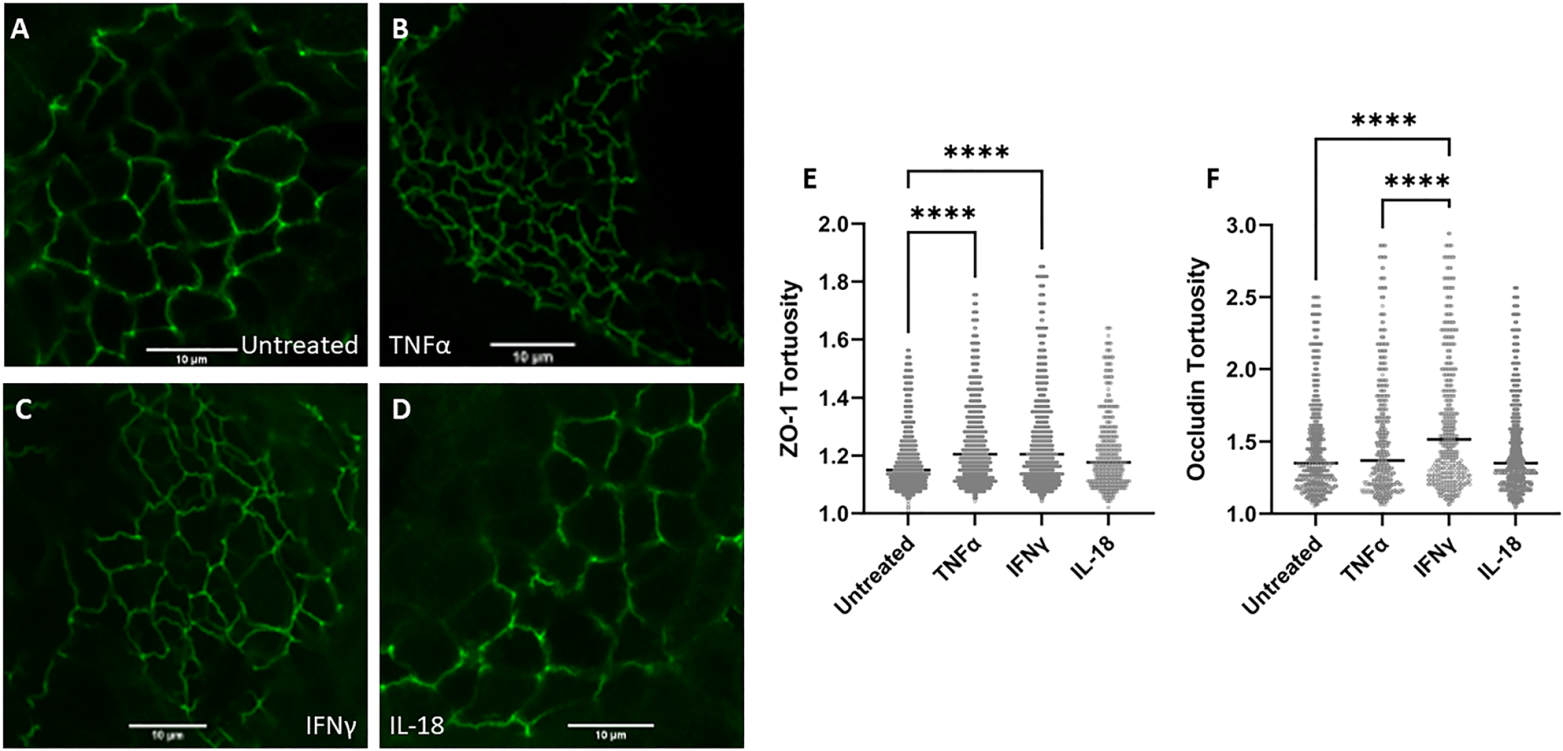
Cytokine treatment alters tight junction tortuosity. (**A–D**) Representative ZO-1 (Alexa Fluor 488) staining of bovine intestinal organoids using a ×100 objective lens following 24-h cytokine treatment. Scale bars denote 10 µm. (**E**) Quantification of ZO-1 tortuosity in cytokine-treated intestinal organoids. TNFα-treated bovine enteroids and IFNγ-treated bovine enteroids displayed a significant increase in junctional tortuosity (p < 0.0001 for each treatment) determined by Kruskal–Wallis non-parametric ANOVA (α = 0.05) utilizing Dunn’s post hoc analysis. (**F**) Quantification of Occludin tortuosity in cytokine-treated intestinal organoids. IFNγ-treated bovine enteroids displayed a significant increase in Occludin tortuosity (p < 0.0001) relative to all other treatments. **** denotes p < 0.0001.

As tight junctions are the primary structure that regulates paracellular movement of water and solutes, we set to determine how inflammatory cytokines impact tight junction structure and spatial conformation. The tortuosity of tight junctions was determined and analyzed through ZO-1 and Occludin immunocytochemistry and image analysis. 24-h treatment of either TNFα or IFNγ induced a significant increase in junctional tortuosity of ZO-1 (p < 0.0001 for each treatment) (Fig. 4). There was no significant effect of IL-18 treatment on junctional tortuosity. 24-h treatment of IFNγ induced a significant increase in Occludin tortuosity (p < 0.0001).

**Figure 5:**
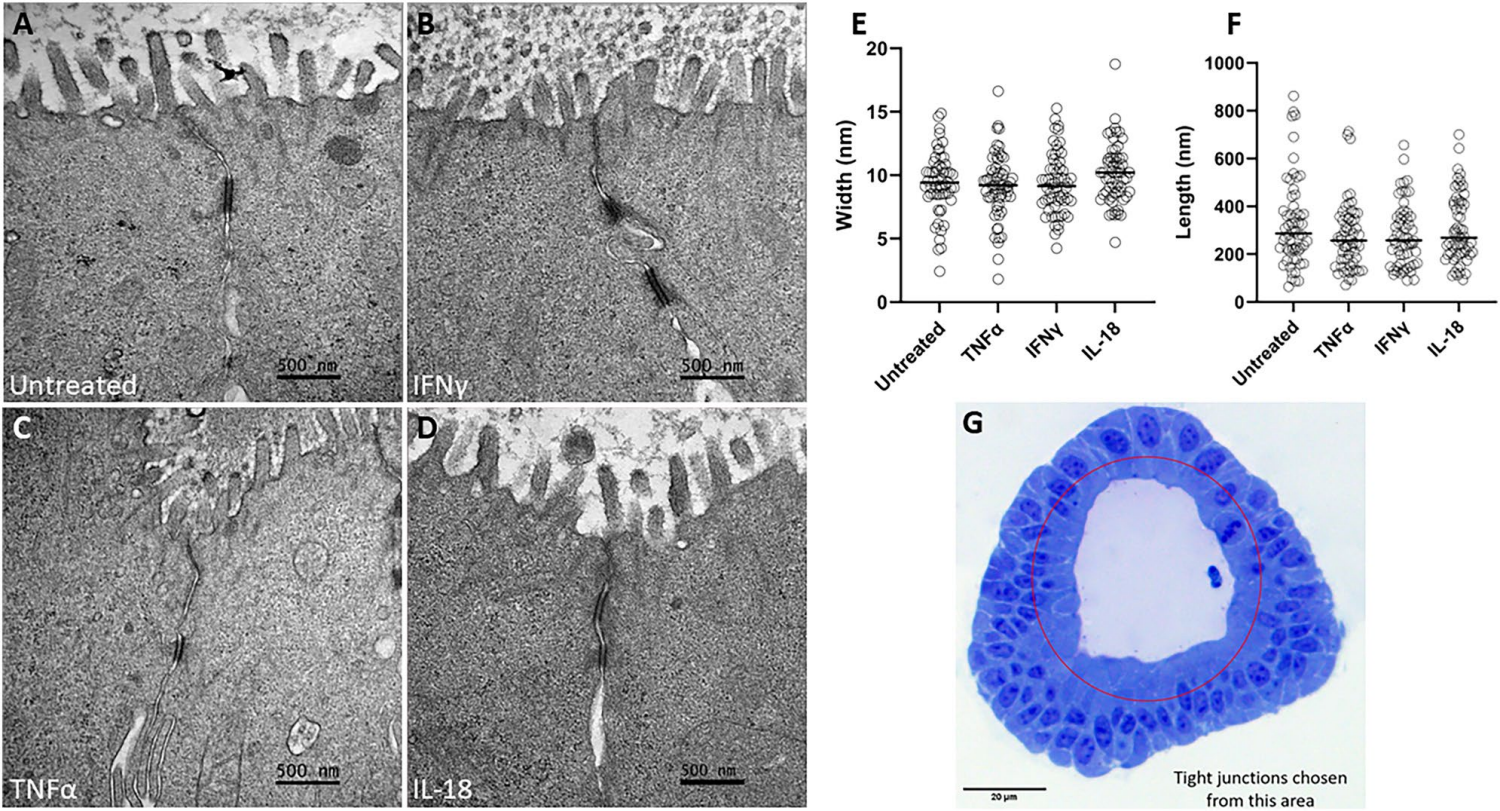
Cytokine treatment does not alter junctional morphology as measured by transmission electron microscopy. (**A–D**) Representative TEM images of cytokine-treated intestinal organoid tight junctions. Scale bars denote 500 nm. (**E**) Tight junction width. (**F**) Tight junction length. (**G**) Light microscopy image of a bovine intestinal organoid highlighting the apical area from which tight junctions were analyzed. Scale bar denotes 20 µm. Cytokine treatment did not induce a significant change in junctional length or width as determined by Kruskal–Wallis non-parametric ANOVA (α = 0.05).

To further investigate the morphology of tight junctions following cytokine treatment, TEM was utilized to measure junctional width and length. Ultrastructure analysis of tight junctions following 24-h cytokine treatment was determined by TEM. Tight junctions were identified by morphology, location, and electron density as guided by previous work^43,44^, and the length width of the tight junctions were measured. No apparent change in tight junction length or width was induced by cytokine treatment (Fig. 5).

### TNFα and IFNγ treatment reduces cellular proliferation in bovine enteroids

**Figure 6:**
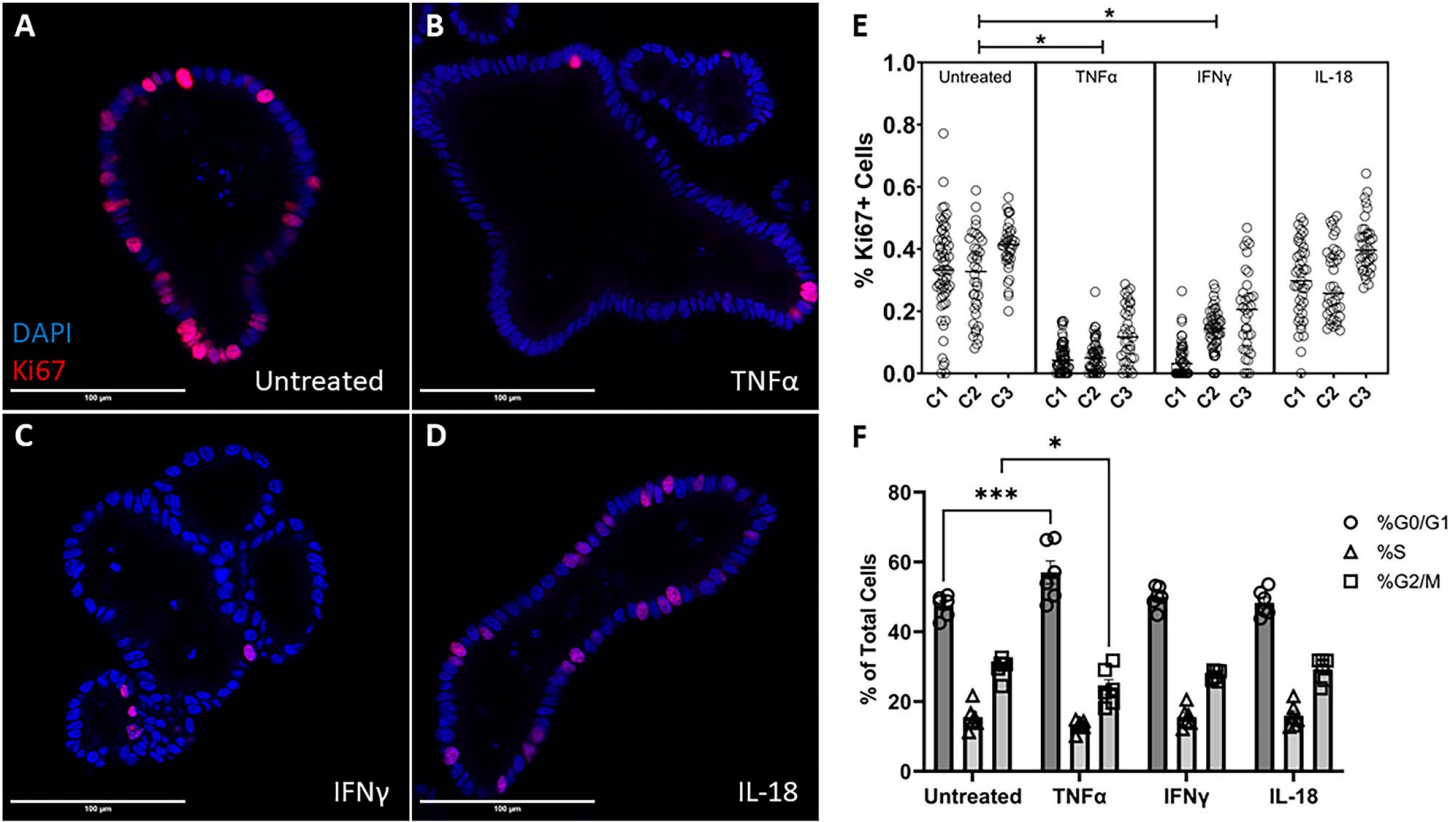
Cytokine treatment alters cellular proliferation. (**A–D**) Representative confocal images of cytokine-treated bovine intestinal organoids stained for Ki67 (Alexa Fluor 594) and DAPI taken on a ×40 objective. Scale bars denote 100 µm. (**E**) Percentage of proliferating cells as measured by confocal microscopy. C1, C2, and C3 indicate individual enteroid lines. (**F**) Cell cycle analysis of cytokine-treated bovine intestinal organoids. TNFα and IFNγ induce a reduction in cellular proliferation (p = 0.0031, p = 0.0072, respectively) evidenced by nested ANOVA (α = 0.05) Holm-Šídák post hoc analysis. Two-way ANOVA displays a significant interaction effect (p = 0.0002) and Dunnett’s multiple comparisons test show that TNFα-treatment induces a rise in the percentage of cells in the G0/G1 phase (p = 0.0003) and a reduction in the percentage of cells in the G2M phase (p = 0.0379). * denotes p < 0.05, *** denotes p < 0.001.

Changes in cellular turnover may lead to junctional morphological changes as cytoskeletal tension may be altered, leading a more severe barrier injury, as suggested by the increase in dextran permeability. To investigate this, we analyzed cellular proliferation in our organoids and measured the proportion of Ki67-positive cells. Treatment with TNFα (100 ng/mL) (0.0703 ± 0.006, Mean ± SEM) or IFNγ (100 ng/mL) (0.121 ± 0.009) significantly reduced the proportion of Ki67-positive cells in bovine enteroids (p = 0.0031, p = 0.0072, respectively) relative to untreated enteroids (0.349 ± 0.01), thus indicating a reduction in cellular proliferation in these treated enteroids (Fig. 6). Enteroids treated with IL-18 (100 ng/mL) (0.3352 ± 0.01) did not exhibit any significant changes in Ki67+ cell fraction.

Cell cycle analysis indicated that TNFα treatment significantly increased the percentage of cells in the G0/G1 phase (p = 0.0003) and significantly decreased the percentage of cells in the G2/M phase (p = 0.0379) relative to untreated enteroids (Fig. 6). IFNγ and IL-18-treated cells did not display a significant change in cell cycle phase percentages.

### IFNγ treatment increases apoptosis in bovine enteroids

Further investigating the effects of cytokine treatment on cellular turnover, we also investigated the apoptotic cell rate in our organoids. IFNγ (100 ng/mL) treatment induced a significant increase in Cleaved Caspase-3 positive cells relative to untreated enteroids (p = 0.0325). TNFα treatment trended towards increasing apoptotic cell rate but was not significant (p = 0.1190). IL-18 treatment did not cause any effect on apoptotic cell rate.

## Discussion

‘Leaky gut’ and downstream metabolic derangements due to intestinal barrier dysfunction are recognized in multiple common diseases of cattle such as rumen acidosis, heat stress, reduced feed intake, and ketosis, though the mechanism of action is poorly understood^17–21^. Inflammation is a shared pathway in all of these pathologies and is known to directly contribute to gut barrier dysfunction in murine models and human intestinal cell lines. Generating comprehensive insight into these cow-specific molecular and cellular pathways may lead to the discovery of novel therapeutics to target the intestinal pathology that coincides with inflammatory diseases. We utilized intestinal organoids to provide us with a physiologically relevant in vitro model capable of extended culture to enable direct investigation of these mechanisms in cattle. By using this model, we have established that the key inflammatory cytokines TNFα and IFNγ, but not IL-18, directly disrupt the intestinal epithelial cell cycle and tight junction conformation, leading to increased barrier permeability. This is the first study that investigates the impact of inflammatory cytokines on gut barrier function in a bovine-specific intestinal epithelium model.

We chose to study the impact of IFN-γ, TNF-α, and IL-18 on gut barrier function in cows because of their relevance to immune function in the gut and their different cellular pathways. Elevated circulating levels of TNFα, a cytokine released by activated immune cells, especially monocytes and macrophages^28^, and IFNγ, a cytokine released by T lymphocytes and specialized immune cells^27^ are associated with bovine inflammatory diseases and accompanying intestinal barrier dysfunction^15,32–35,45^. IL-18, on the other hand, is produced by the epithelial cells themselves, and its primary function is to stimulate immune cells and induce downstream release of proinflammatory cytokines^46^.

IFN-γ and TNF-α treatments, but not IL-18, disrupted intestinal barrier function as evident by the increased movement of 4 kDa-dextran across the epithelial surface (Fig. 3G). A larger, 70 kDa-dextran displayed a mitigated affect compared to the 4 kDa dextran (Fig. 3G,H), showing reduced permeability to larger molecules. Accompanying the increase in permeability were morphological changes including luminal darkening and shedding of cells (Fig. 2). Our findings are consistent with previous studies in other species’ organoids or cell lines^12,47,48^, but extend upon these previous findings to show the effects of specifically treating bovine intestinal epithelium with bovine inflammatory cytokines via an organoid system. Maintaining an effective barrier between the basolateral and apical sides of the epithelium is a critical role of the intestinal epithelium, and the associated change to barrier permeability accompanying cytokine treatment indicates damage to and reduced function of the intestinal epithelial barrier.

Given the significance of tight junctions in maintaining the barrier function and the deleterious effects of IFNγ and TNFα identified in human and non-human animal cell lines^49–55^, we hypothesized that IFNγ and TNFα increase intestinal epithelial permeability in cattle via the disruption of tight junctions. Tight junctions are critical structures in the regulation of paracellular permeability of small molecules, and exposure to cytotoxic compounds alters tight junctions and increases paracellular permeability^56,57^. Downregulation of barrier-relevant tight junction proteins does not coincide with infection-induced barrier disruption in cultured epithelial cells^58^. However, the tortuosity of epithelial tight junctions is a measurable morphological trait that does coincide with a disrupted intestinal barrier^59^. Furthermore, increased cellular proliferation or inhibition of Rho kinase, and consequent inhibition of apoptosis, decrease the tortuosity in MDCK epithelial cell tight junctions^60^. Therefore, we have investigated tight junction conformation, specifically tortuosity, to further elucidate the mechanisms of permeability alteration observed in our study. Our findings show that treatment of bovine intestinal organoids with either TNFα or IFNγ does increase tight junctional tortuosity (Fig. 4), consistent with tortuosity changes seen in other models of barrier disruption^59,60^, supporting a mechanistic role of tight junction conformation in bovine inflammation-induced gut barrier dysfunction. Despite the rise in tortuosity caused by cytokine treatment, there was no discernible difference in tight junction length or width, as measured by electron microscopy coinciding with cytokine treatment (Fig. 5).

**Figure 7:**
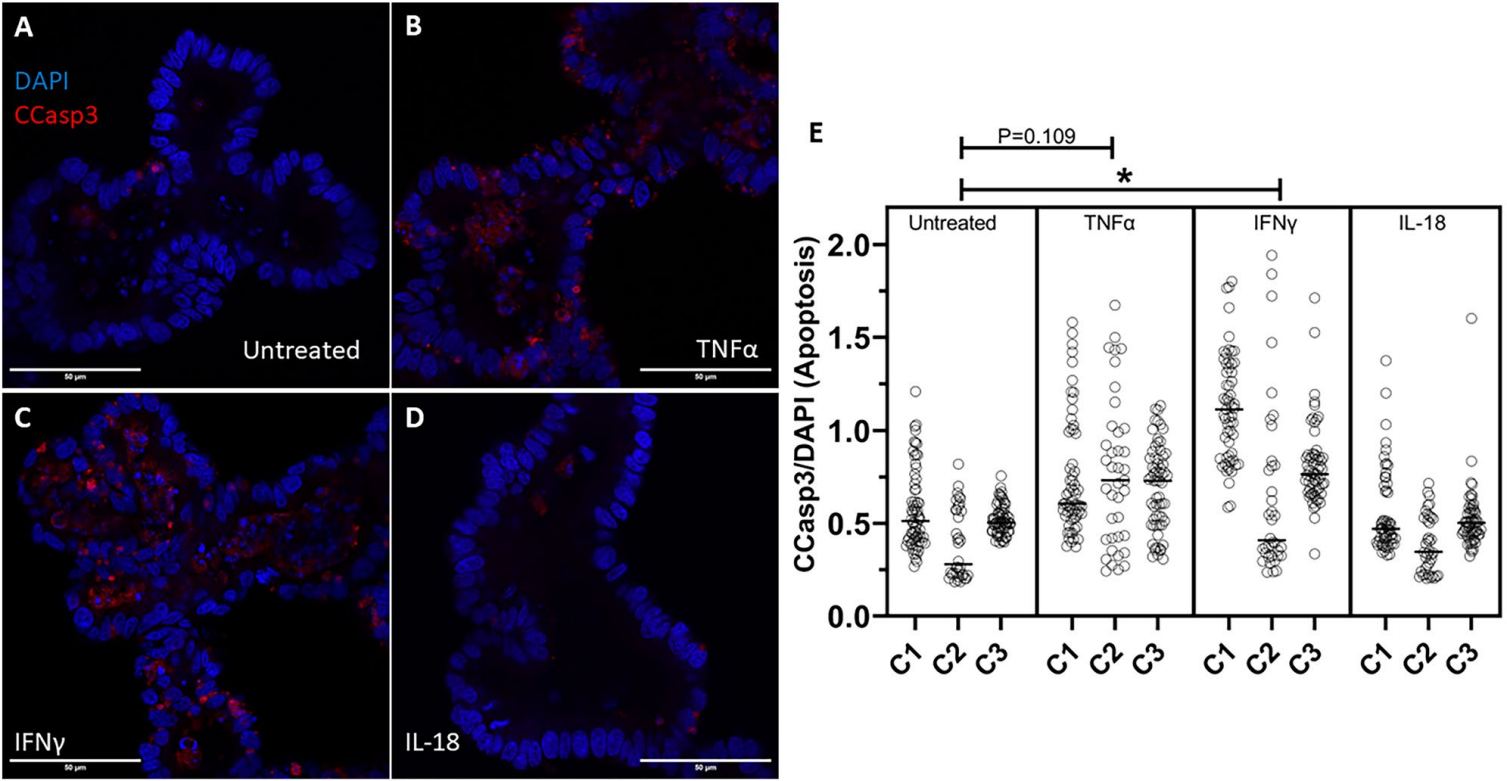
Cytokine treatment alters apoptosis. (**A–D**) Representative confocal images of cytokine-treated bovine intestinal organoids stained for Cleaved Caspase-3 (CCasp3) (Alexa Fluor 594) and DAPI using a ×63 objective lens. Scale bars denote 50 µm. (**E**) Area of intraluminal CCasp3 normalized to DAPI measured by confocal microscopy. C1, C2, and C3 indicate individual enteroid lines. IFNγ induces a rise in apoptotic cells (p = 0.0325) determined by nested ANOVA Holm-Šídák post hoc analysis.

While the increased movement of molecules across the paracellular pathway due to disrupted tight junctions can explain the increased gut permeability noted in vivo and in our in vitro model, we wanted to further investigate the role of cellular integrity and turnover as a potential additional factor. A healthy intestinal epithelium undergoes immense cellular turnover and completely regenerates over the course of four to five days^23^, and excessive cell death may lead to barrier dysfunction and translocation of pathogens^61^. Moreover, inhibition of apoptosis ameliorates barrier dysfunction induced by *C. jejuni* infection in cultured HT-29/B6-GR/MR epithelial cells^58^. We hypothesized that cytokine treatment would negatively affect cellular turnover by reducing cellular proliferation and stimulating cell death. Treatment of bovine intestinal organoids with either TNFα or IFNγ reduced cellular proliferation as measured through immunocytochemical staining, and cell cycle analysis displayed an increase of cells in the G0/G1 phase and a reduction of cells in the G2/M phase when the organoids were treated with TNFα (Fig. 6). Furthermore, IFNγ treatment also induced a rise in apoptosis (Fig. 7). Altogether, whether by reducing proliferation via TNFα or IFNγ or stimulating apoptosis via IFNγ, inflammatory cytokines disrupt the cellular turnover of the epithelium in bovine intestinal organoids thus influencing the replacement of lost epithelial tissue. Our findings corroborate others that show a rise in apoptosis following cytokine or infection-induced barrier dysfunction^62–64^. One potential mechanism for the phenomena observed in our study could involve the activation of Rho GTPase via TNFα and IFNγ, which causes an increase in cell extrusions and the persistence of single cell lesions^64,65^, and Rho kinase activation alters cytoskeletal forces and causes an increase in tight junctional tortuosity^60^. Reduced cellular turnover resulting in an altered tension on cytoskeletal actin filaments could explain the causative mechanism of increased permeability seen in our bovine intestinal organoids and would be sensible considering the reduced cellular proliferation and increased cellular death shown in our study.

Our study is not without limitations. While our organoid culture system does improve on the physiological relevance and translatability to native intestine compared to traditional cell line culture, the organoids are comprised of only intestinal epithelial cells and do not include other cell types seen in the gut such as professional immune cells and smooth muscle cells. Additionally, though our cytokine treatments are consistent with other published in-vitro treatments^66–69^ at supraphysiologic concentrations and for relatively short periods of time, unlike chronic inflammatory conditions in-vivo which result in lower concentrations of cytokines for far longer time frames.

In conclusion, these data show that treatment with the inflammatory cytokines, TNFα or IFNγ, directly disrupts the epithelial barrier, leads to more tortuous tight junctions, and alters cellular turnover in bovine intestinal organoids. These effects were not observed when intestinal organoids were treated with IL-18. These findings further clarify the influence of systemic inflammation on the bovine gut and expand on the role of bovine intestinal organoids as a model to investigate the bovine gut and related pathologies (Supplementary information).

## Supplementary Information

Supplementary Information.

## Data Availability

All raw data used to generate figures are provided as source data with this paper. All images generated for this study are available from the corresponding author upon request.
